# An open database of computed bulk ternary transition metal dichalcogenides

**DOI:** 10.1038/s41597-023-02103-4

**Published:** 2023-05-30

**Authors:** Scott E. Muller, Micah P. Prange, Zexi Lu, W. Steven Rosenthal, Jenna A. Bilbrey

**Affiliations:** Pacific Northwest National Laboratory, Richland, WA 99352 USA

**Keywords:** Atomistic models, Structural properties, Electronic properties and materials, Electronic structure, Structure prediction

## Abstract

We present a dataset of structural relaxations of bulk ternary transition metal dichalcogenides (TMDs) computed via plane-wave density functional theory (DFT). We examined combinations of up to two chalcogenides with seven transition metals from groups 4–6 in octahedral (1T) or trigonal prismatic (2H) coordination. The full dataset consists of 672 unique stoichiometries, with a total of 50,337 individual configurations generated during structural relaxation. Our motivations for building this dataset are (1) to develop a training set for the generation of machine and deep learning models and (2) to obtain structural minima over a range of stoichiometries to support future electronic analyses. We provide the dataset as individual VASP xml files as well as all configurations encountered during relaxations collated into an ASE database with the corresponding total energy and atomic forces. In this report, we discuss the dataset in more detail and highlight interesting structural and electronic features of the relaxed structures.

## Background & Summary

Transition metal dichalcogenides (TMDs) have fascinating electrical, optical, and mechanical properties^1,2^, with wide-ranging applications in sensing^3^, electronics^4^, batteries^5^, catalysis^6,7^, and pollution remediation^8^. The structure and properties of TMDs depend on the choice of metal and chalcogenide, as well as the crystal structure. Combinations of chalcogenides and/or metals lead to different behaviors that allow TMDs to be tailored for a desired structural phase or band gap^9^. The number of potential metal/chalcogenide combinations is challenging and expensive to explore by synthesis and experiment. The lack of data covering the full range of possible stoichiometries hinders data-driven scientific discovery. Computational modeling of TMD structures and calculation of their resulting properties will allow for downselection of potential TMD combinations for synthesis.

Pure TMDs have a stoichiometry of MX_2_, where M is a transition metal and X is a chalcogenide. Ternary and quaternary TMDs, containing mixtures of metals and/or chalcogenides, have been investigated for improved structure and property control. For example, Zhou *et al*. synthesized 13 TMD alloys (11 ternary, 1 quaternary, and 1 quinary) via molten-salt-assisted CVD^10^. Susarla *et al*. showed that the bandgap of quaternary alloys of Mo_*γ*_W_1−*γ*_S_2*δ*_Se_2(1−*δ*)_ could be tuned between 1.61 and 1.85 eV, with theoretical investigation demonstrating tuning between an even wider range of 1.60 and 2.03 eV^11^.

**Fig. 1:**
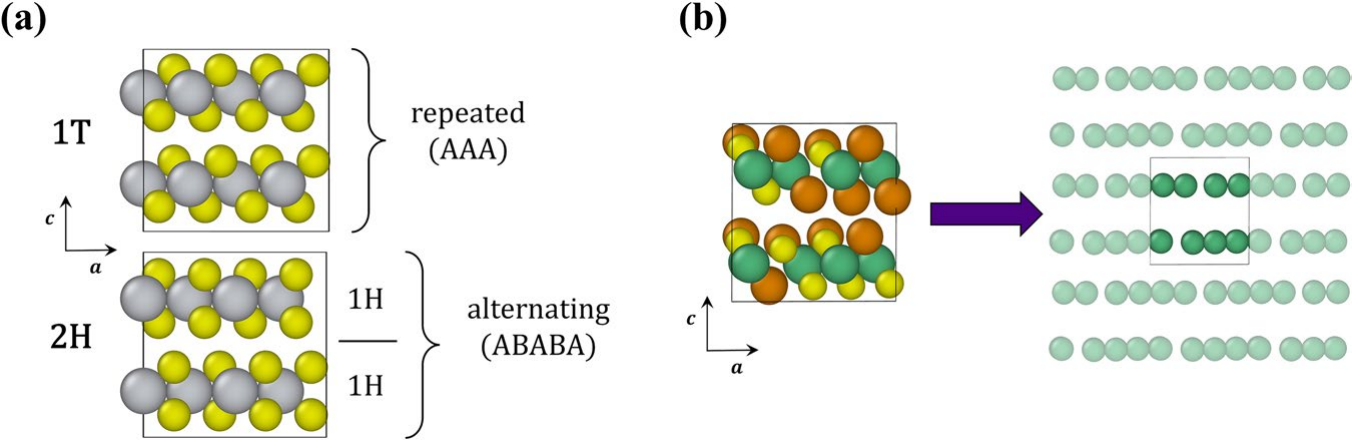
Details of structure and composition for studied TMDs. (**a**) Structural depictions of 1T and 2H TMDs, where gray spheres represent a transition metal and yellow spheres represent a chalcogenide. (**b**) Structural depiction of a 1T′ distorted structure, where green spheres represent a transition metal and yellow and orange spheres represent different chalcogenides. At the right we show the unit cell (inside the box) and its semi-transparent periodic images, with chalcogenides removed to show the broken symmetry in the 1T′ polytype.

In addition to the large variety of possible elemental compositions, TMDs are polymorphic and thus form several polytypes distinguished by intralayer coordination and interlayer stacking. Here, we focus on the 1T and 2H polytypes, where the number denotes the number of layers present in a repeating unit, and the letter denotes the crystal system (tetragonal or hexagonal) with the transition metal sitting in either octahedral (1T) or trigonal prismatic (2H) sites^12^. These are shown in Fig. 1a. The relative stabilities of polymorphs vary across the TMD family, with the primary influence on stability being the variance in *d* orbital electrons depending on the constituent transition metal group in the periodic table; e.g., TMDs with group 4 and 6 metals often present in the 2H phase, while those with group 5 metals present as both 1T and 2H^13^. Metastable polytypes can be stabilized through various techniques, including the intercalation of ions between the van der Waals layers and reaction quenching^14,15^.

To add to the complexity of these systems, distorted polytypes resulting from intralayer symmetry breaking have also been observed experimentally. Lu *et al*. attributed the broken symmetry in 1T′ WTe_2_ to charge density wave distortion, which causes the vertical displacement of transition metal atoms and is clearly observed along the [100] and [010] orientations^16^, demonstrated in Fig. 1b. Lai *et al*. observed a similar variation in M-M distances in 1T′ WS_2_ structures^17^. The distorted 1T′ phase of MoTe_2_—a semimetal with superconductivity—has been formed through laser-induced and thermal phase transitions^18–21^, and synthesized via chemical vapor deposition (CVD)^22,23^ and molecular beam epitaxy (MBE)^24^. Zhang *et al*. found that a metal-to-insulator transition can be induced in W_*γ*_Mo_1–*γ*_Te_2_ under ambient conditions resulting from a transition from the 2H (semiconducting) to 1T′ (semimetallic) polytype, which demonstrates the correspondence between structural and electronic properties in these systems^25^.

The electronic and magnetic properties of TMDs are dependent on the composition, which can be studied by examining the variation in the computed electronic structure induced by changing the identity and concentrations of the atomic constituents. Many of the desirable properties in this class of materials arise from the transition metal *d* orbitals. The number and behavior of electrons occupying these orbitals are affected by the identity of the transition metal, with more *d* orbitals filled as one moves right on the periodic table. They are also affected by the chalcogen species, which interact with the transition metal through the valence *p* orbitals. These orbitals grow in size with the principal quantum number n. There is still a need for thorough computational analyses across a broad compositional space to understand underlying trends in these materials.

The diverse composition and varied structural and electronic properties make TMDs ripe for machine learning (ML) analysis. Sumi *et al*. used ML to predict the semiconductor-to-metal transition in MoX_2−*δ*_O_*δ*_ bilayers (where X is a chalcogenide) and found that the concentration of chalcogen atoms in the interlayer van der Waals region was a leading predictor of the semiconductor-to-metal transition^26^. Zhao *et al*. developed a high-throughput screening approach combining first-principles computations with ML to screen for synthesizable 2D TMDs with effective Hg^0^ sensing response^27^. Their approach relied on the use of AFLOW-ML, a software tool to apply ML-based property prediction algorithms to the user’s dataset of interest^28^. Behler discusses the acceleration of atomic-scale simulations of condensed systems using neural network potentials (NNPs), noting the need for electronic structure data computed at high level of theory to serve as training sets^29^. For any ML approach, particularly neural networks, it is crucial that sufficient data is available for training, testing, and validation^30^.

The field of computational materials science has seen rapid advancements due to the availability of large datasets generated using high-throughput calculation methods and/or supercomputing resources. In particular, the National Institute of Standards and Technology (NIST)’s wide-spanning Materials Genome Initiative (MGI)^31^, and the associated Materials Project^32^, have laid the foundation for researchers to share simulation results in a way that supports FAIR data principles – Findability, Accessibility, Interoperability, and Reusability^33^. Repositories with strict computational requirements or more specialized information, such as the joint automated repository for various integrated simulations (JARVIS)^34^, are also available.

Supervised ML approaches, commonly applied in the materials science domain, require training datasets that encompass the configuration space of interest. Meanwhile, tuning the performance of TMD-based devices via constituent composition requires knowledge of the relationship among compositional variables, atomic structure, and functional properties. To support both needs, we produced a database of structurally relaxed ternary TMDs computed at the DFT level. We examined 1T and 2H ternary structures (MX_2–*δ*_Y_*δ*_) of seven transition metals (M = Ti, V, Nb, Mo, Hf, Ta, and W) with varying binary combinations of chalcogenides (X, Y = S, Se, and Te), comprising 672 unique stoichiometries. For a subset of the computed structures, we provide spin-polarization calculations to characterize their electronic structure. Our primary motivations for building this dataset are (1) to provide a rich dataset for training ML-based property prediction models and NNPs and (2) to obtain structural minima over a range of stoichiometries for further electronic analyses. In this report, we discuss the computational methods used to compute the structures and provide brief structural and energetic analyses of the computed ternary TMDs. The database and corresponding analysis scripts are openly available at 10.6084/m9.figshare.21308157.

## Methods

### Composition selection

We consider ternary TMDs with the stoichiometry MX_2-*δ*_ Y_δ_, where *δ* is the ratio of Y atoms to M atoms. We include the chalcogens S, Se, and Te, along with seven transition metals from three different columns in the periodic table: two metals are from group 4 (Ti, Hf), three metals are from group 5 (V, Nb, Ta), and two metals are from group 6 (Mo, W). These transition metal groups tend to be stable in TMDs at ambient pressure and temperature^35^. We select only metals that are generally stable with all three chalcogenides, which precludes Cr from group 6^36^. We also neglect Zr, as TMDs from group 4 follow similar trends in behavior, and we already include the corresponding lighter and heavier metals (Ti and Hf, respectively)^37^.

We initialize each TMD as either the 1T or 2H polytype. While in binary form the 1T structure can be effectively simulated as a single layer, ternary TMDs will almost certainly break the symmetry that can be represented by one layer. Thus, we consider two-layered TMD simulation cells for both 1T and 2H structures. Note that finite size effects still exist due to artificial symmetry imposed by periodic boundary conditions of the simulation cells. Our TMD simulation cells consist of 48 atoms, with 24 atoms in each layer. The 2H structures use the same *c* lattice parameter as in their primitive unit cell, while the *c* lattice parameter is doubled for the 1T structures. From the primitive 2-layer structure, which consists of 6 atoms, our 48-atom simulation cell is formed by quadrupling the primitive cell in both the *a* and *b* directions, and then partitioning a nearly orthorhombic section of 48 atoms. This yields a simulation cell with dimensions $a=\left(2\sqrt{3}\right){a}_{0}$, *b* = 2*a*_0_, and *c*_1T_ = 2*c*_0_, and *c*_2H_ = *c*_0_, where *a*_0_ and *c*_0_ are the primitive lattice parameters. Chalcogenide lattice site occupations for mixed TMD structures are chosen in the manner of special quasirandom structures^38^. Note that there are many ways that specific chalcogenides may be distributed to lattice sites in mixed TMDs—a good review of structured TMDs was compiled by Zhang *et al*.^39^ The chalcogenide stoichiometric ratio *δ* ranges from 0 to 2 with minimum steps of 0.25 (corresponding to the replacement of 4 chalcogenide atoms in the simulation cell). For the 1T polytypes, we examine a reduced step size of 0.0625 (corresponding to the replacement of 1 chalcogenide atom in the simulation cell) from 0 to 0.5 and 1.5 to 2 to explore fine-grained effects of chalcogenide mixing.

### Simulation specifics

**Fig. 2:**
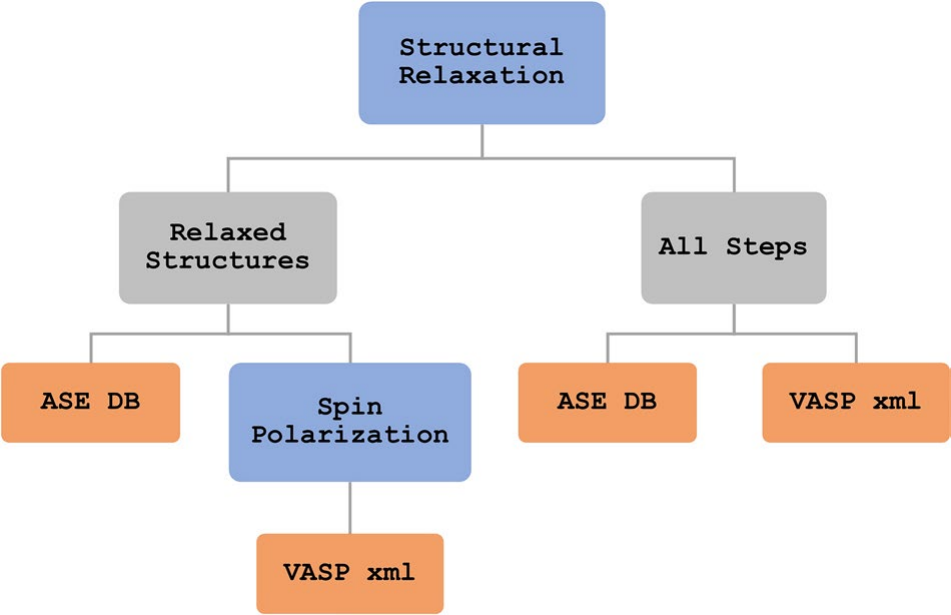
Simulation workflow. Methods are in blue, outputs in gray, and data products in orange.

We carry out positional relaxation of TMD structures using the Vienna Ab-initio Simulation Package (VASP)^40,41^, which solves the Kohn-Sham equations of density functional theory using plane-wave basis sets (defined by a 600 eV cutoff) in a periodic system. A conjugate gradient algorithm is applied to optimize structural parameters, including the internal coordinates and the cell vectors. We use the generalized gradient approximation (GGA) of the Perdew-Burke-Ernzerhof (PBE) functional^42^ to compute the exchange-correlation potential. The projector augmented wave (PAW) method was used with parameters included in the VASP supplied POTCAR files. These exhibited 4 (Ti, Hf), 5 (V, Ta), or 6 (S, Se, Te, Mo, W) explicit electrons per atom. Nb exhibited 11 explicit electrons per atom because it also included the 4p^6^ electrons in the valence electrons. Relaxations were performed with a Gamma-centered 3 × 6 × 4 Monkhorst-Pack grid to sample the Brillouin zone. Orbital occupancies were broadened with Gaussian smearing with a width of 0.01 eV. We include van der Waals corrections with zero damping using Grimme *et al*.’s DFT-D3 method^43^. We consider a structure to be in a relaxed state when the magnitude of the maximum force on any atom is less than 0.01 eV/Å. All input parameters for the VASP calculations can be retrieved from the datafiles supplied in MXY_DB, with the simulation workflow shown in Fig. 2. For most δ values only a single supercell was generated. An exception was *δ* = 1.0 where two supercells were generated for each structure, with the initial chalcogenide locations being identical but the chalcogenide types reversed. We compared our quasirandom structures to a large set of structures (200) with completely randomly assigned chalcogenides for specific *δ* values and found that the energy of our original quasirandom structures were within a standard deviation of the mean energy value, thus representing a typical structure.

Spin-polarization calculations were carried out on a subset of the relaxed structures (316 total). We perform collinear spin-polarized calculations within the PBE + *U* formalism^44^, where the semi-local approximate DFT exchange-correlation potential is corrected with onsite Hubbard terms that cancel the tendency of GGA to over-delocalize electrons. Parameters for the *U* correction are taken from the Hubbard *U* (Atomic) values obtained by Kirchner-Hall *et al*.^45^. Due to the wide variety of electronic structures observed, a complete analysis of all the variation is beyond the scope of this manuscript, but could be undertaken with the data provided in MXY_DB.

### Property calculations

Along with the atomic coordinates, total energy (*E*_0_), and atomic forces, we also conduct analysis on some structural and energetic features for each relaxed structure. Structural features include interlayer spacing, cell lengths (*a*, *b*, *c*) and angles. Energetic features include per-atom formation energy (*E*_form_), calculated as

1 $${E}_{{\rm{form}}}=\frac{1}{3{N}_{{\rm{M}}}}\left\{{E}_{0}\left({{\rm{MX}}}_{2-\delta }{{\rm{Y}}}_{\delta }\right)-{N}_{{\rm{M}}}\left[{E}_{{\rm{M}}}+\left(2-\delta \right){E}_{{\rm{X}}}+\delta {E}_{{\rm{Y}}}\right]\right\},$$

where *E*_M_, *E*_X_, and *E*_Y_ are the per-atom energies of the elemental solid phases of the metal M and chalcogens X and Y and *N*_M_ is the number of metal atoms in the unit cell (16 in our case). We obtain monoelemental structures from the Materials Project database^32^ and relax them under the same approximations as used for relaxation of the ternary TMDs.

Binary TMDs have been experimentally observed with both octahedral (1T) and distorted octahedral (1T′) coordination. To determine which polytype should be used in the calculation of *E*_form_, we compute the energy-per-atom for binary structures with primitive 1T and 1T′ unit cells, which lack the degrees of freedom to change phase during structural relaxations. The results of these calculations are shown in Table 1. The polytype with lower *E*_form_ is considered to be more stable for a given binary TMD. Binary TMDs with group 4 metals (Ti, Hf) were found to be more stable in the 1T polytype, while group 6 metals (Mo, W) produced greater stability with the 1T′ polytype. The stability of binary TMDs with group 5 metals (V, Nb, Ta) was dependent on the chalcogenide present: 1T was more stable with S or Se and 1T′ was more stable with Te. We note that the energy difference is small in many cases, indicating some degree of degeneracy in the polytype for TMD structures. We use the most stable structure to determine the initial pre-relaxed atomic arrangement for binary TMDs: when *E*(1T)>*E*(1T′), we use the 1T′ polytype in the calculation of *E*_form_, otherwise we use 1T.

**Table 1:** Energy of formation from elements of 1T and 1T′ polytypes for binary TMDs.

		Energies in eV/atom		
		*E*_form_ (1T)	*E*_form_ (1T′)	Δ*E*_form_ (1T→1T′)
Group 4	TiS_2_	−6.798	−6.791	0.006
	TiSe_2_	−6.268	−6.259	0.009
	TiTe_2_	−5.646	−5.632	0.014
	HfS_2_	−7.855	−7.851	0.003
	HfSe_2_	−7.244	−7.240	0.004
	HfTe_2_	−6.533	−6.521	0.012
Group 5	VS_2_	−6.779	−6.777	0.002
	VSe_2_	−6.237	−6.205	0.032
	VTe_2_	−5.676	−5.706	−**0.030**
	NbS_2_	−7.358	−7.354	0.004
	NbSe_2_	−6.815	−6.812	0.003
	NbTe_2_	−6.211	−6.229	−**0.017**
	TaS_2_	−7.971	−7.970	0.001
	TaSe_2_	−7.354	−7.353	0.001
	TaTe_2_	−6.698	−6.736	−**0.038**
Group 6	MoS_2_	−7.246	−7.333	−**0.087**
	MoSe_2_	−6.721	−6.834	−**0.113**
	MoTe_2_	−6.165	−6.317	−**0.151**
	WS_2_	−7.854	−7.965	−**0.111**
	WSe_2_	−7.288	−7.441	−**0.153**
	WTe_2_	−6.650	−6.859	−**0.209**

The rightmost column is given by Δ*E*_form_(1T→1T′) = *E*_form_(1T′)−*E*_form_(1T); text in this column is bolded for binary TMDs with better 1T′ stability.

## Data Records

We distribute MXY_DB via Figshare^46^. Datasets include VASP xml files for the individual structural relaxations, VASP xml files for the spin-polarized calculations, and SQLite3 databases containing all structures of a given polytype, including intermediate steps taken during structural relaxation, generated using the atomic simulation environment (ASE) python library^47^. Each row in the database is composed of an ASE Atoms object, which has information on the elements, coordinates, cell, and periodic boundary conditions, along with a dictionary containing the total energy (eV) and forces on each atom (eV/Å). Python scripts used to collate the database and perform structural analysis are provided alongside the data.

**Fig. 3:**
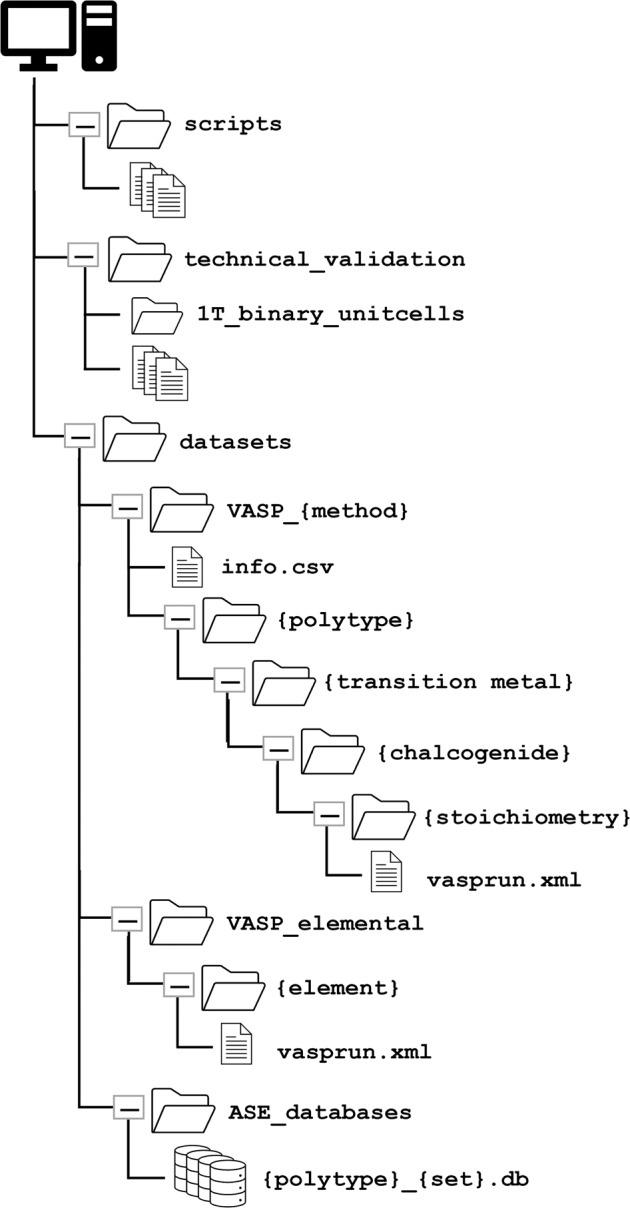
File structure of MXY_DB.

The file structure of MXY_DB is shown in Fig. 3. All the datasets are contained in the datasets folder. The simulation output is organized by calculation method (structural_relaxation or spin_polarization), polytype (1T or 2H), transition metal (Ti, V, Nb, Mo, Hf, Ta, or W), chalcogenide(s) (S, Se, Te, SSe, STe, or SeTe), and stoichiometry of the lowest-Z chalcogenide (numerical value). Each calculation method folder has a table containing information on the polytype, elements, stoichiometry, relative file path, and calculated properties to allow for search and filtering of the various calculations. VASP xml files for per-atom energies of the elemental solid phases used to compute *E*_form_ are provided in the VASP_elemental folder, which is organized by element (Ti, V, Nb, Mo, Hf, Ta, W, S, Se, or Te). Four distinct ASE databases are in the ASE_databases folder: minimized structures for 1T or 2H ({polytype}_relaxed.db) and all structures generated during relaxation for 1T or 2H ({polytype}_all_steps.db). Python-based scripts used to generate the databases and analyze simulation data are in the scripts folder, along with a README.md describing each script. Information supporting our validation of the database, including unit cell computations for 1T and 1T′ binary TMDs and experimental lattice parameters and densities, is included in the technical_validaiton folder.

## Technical Validation

**Fig. 4:**
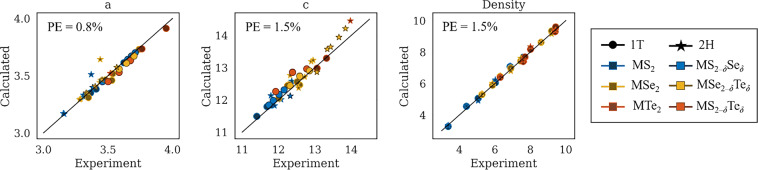
Comparison between experimental and computed parameters. These include lattice parameters *a* and *c* (Å) and density (g/cc) going from left to right. Percent error (PE) is shown in the upper left of each plot. Lattice parameters for 37 TMDs (16 ternary, 21 binary)^48,49^ and densities of 20 TMDs (all binary)^17,50^ were obtained from the literature (some by computation using lattice parameters). In instances where the literature value is reported for a single layer, *c* is doubled to compare with our two-layer cells, and where the polytype was not reported, we plot against calculated values for both polytypes.

To validate our computed binary (MX_2_) and ternary (MX_2–*δ*_Y_*δ*_) structures, we compare lattice parameters *a* and *c* and density against experimental values found in the literature. Experimental lattice parameters were obtained for 37 TMDs, 16 of which are ternary structures^48,49^. Comparisons between the experimental values and those arising from our relaxed structures are shown in Fig. 4. Note that we doubled the reported *c* values for 1T polytype structures to comparing to our 2-layer structures. Correlation between the experimental lattice parameters and our computed lattice parameters is very high, with percent errors of 0.8% and 1.5% and mean absolute errors (MAE) of 0.028 and 0.147 Å for *a* and *c*, respectively. While the MAE of *a* is the same for both 1T and 2H polytypes, the MAE of *c* is much lower for the 1T polytype: 0.070 versus 0.237 Å for 1T and 2H polytypes, respectively. Values for the experimental and computed primitive lattice parameters *a* and *c* for various structures in the database are given in MXY_DB. The primitive lattice parameters for all structures in MXY_DB are given in the info.csv file of the VASP_structural_relaxation dataset.

The densities of 14 binary TMDs were obtained from the CRC Handbook of Chemistry and Physics^50^, which didn’t report polytype with their structures. We computed an additional 6 experimental densities using the 1T polytype lattice parameters reported by Lai *et al*.^17^ with their corresponding stoichiometries. The only binary TMD structures for which we found no experimental density were VS_2_ and VTe_2_. Figure 4 shows the density comparison between experimental and the corresponding relaxed binary structures; values for the experimental and computed densities (g/cc) are given in MXY_DB. No experimental density values for the ternary TMDs included in MXY_DB could be found in the literature. In addition, not all reported densities were associated with a polytype; therefore, we compare the reported value against both polytypes in the figure. Our computed values match well with experimental values, showing a MAE of 0.104 g/cc and MSE of 0.016 g/cc.

**Fig. 5:**
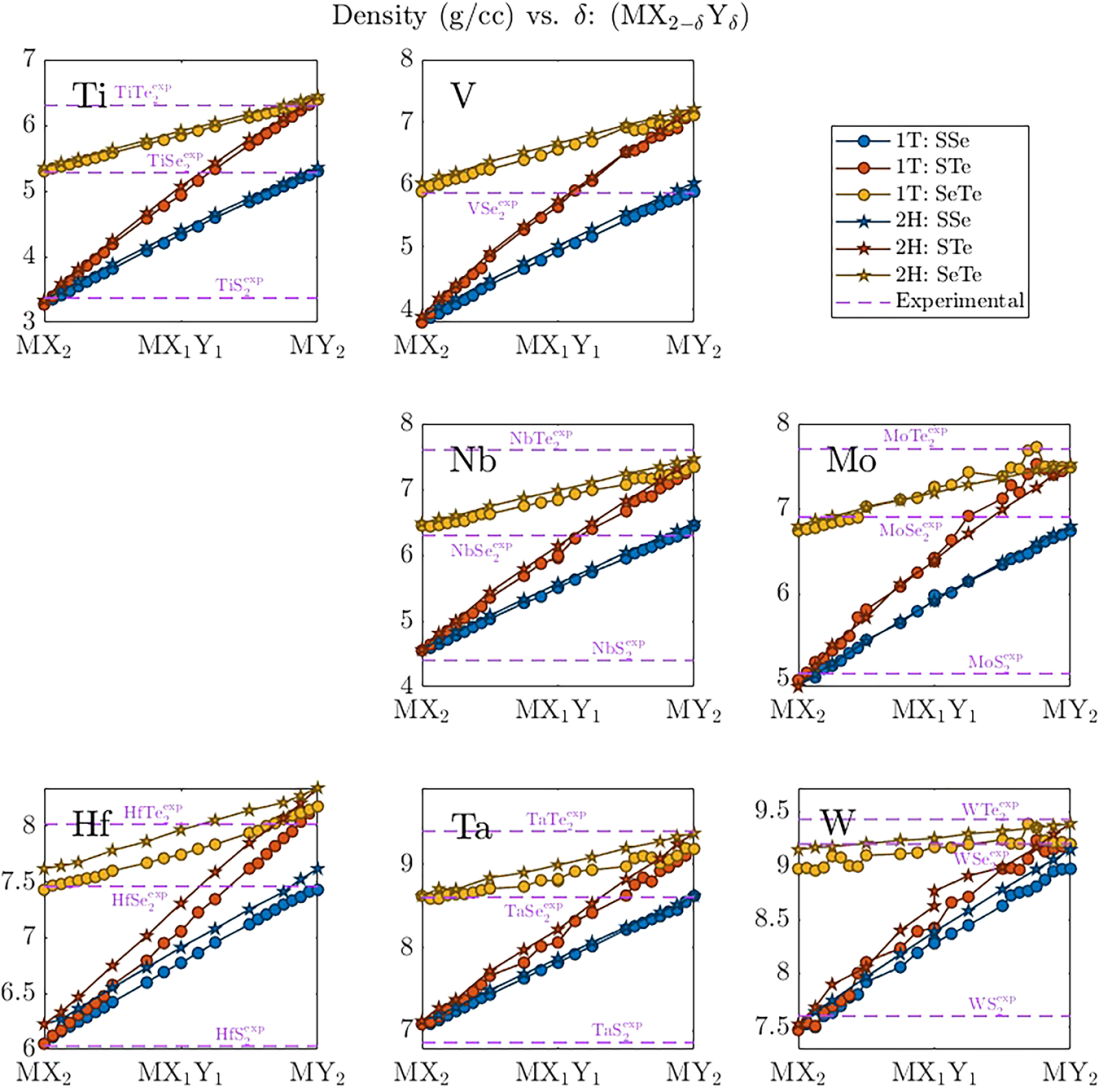
Computed densities of ternary TMDs. Density is in units of g/cc. Comparison is made to available experimental values (pink dotted lines, see MXY_DB for more details)^17,50^. The location of each plot corresponds to the location of the transition metal on the periodic table.

As the X:Y ratio in ternary TMDs increases, a linear change in density from that of MX_2_ to that of MY_2_ is expected. As shown in Fig. 5, the expected trend is observed for all metal-chalcogenide-polytype combinations examined in MXY_DB. Slight deviations from linearity for individual structures are more frequent for structures containing group 6 metals (Mo and W). These deviations are the result of increased symmetry breaking during structural relaxations, leading to several local minima, and demonstrates the complicated dependency of structural properties on chalcogenide stoichiometry. In general, the high correlation between available experimental and our computed structural parameters, along with the linear transition in computed densities, indicates the quality of the structures in MXY_TMD.

## Usage Notes

VASP xml files can be read using common Python packages such as ASE (ase.io.read(‘vasprun.xml’)) and pymatgen (pymatgen.io.vasp.outputs.Vasprun(‘vasprun.xml’)), as well as visualization programs such as JMol and VMD. ASE databases can be read via ase.db.connect(‘{polytype}_{set}.db’). ASE Atoms objects can be converted to pymatgen Structure objects using pymatgen.io.ase.AseAtomsAdaptor.

**Fig. 6:**
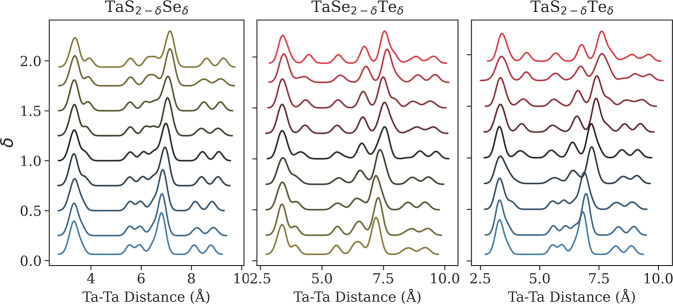
Radial distance function (RDF) of M-M distances for Ta-series ternary TMDs. The color gradient corresponds to *δ*, where blue represents S, yellow Se, and red Te.

The computational results provided in MXY_DB can be used to examine structural changes, such as lattice distortions, stacking symmetry, interlayer distances, and distortion in the 1T polytype, arising from chalcogenide substitutions. For instance, stability of the 1T′ phase is desired in order to manifest useful optical and electronic properties resulting from reduced symmetry in the distorted phase^15^. Phase classification is based on symmetries of the crystallographic cell, which are broken by the quasirandom substitution of chalcogenides in ternary TMDs. Ternary TMDs, instead, take on intermediate phases in high-dimensional space. Therefore, instead of direct classification of the phase, we identify the amount of distortion in the transition metal (M) lattice through examination of the radial distance function (RDF) of M-M distances, which provides a 1D descriptor of polytype distortion. Figure 6 shows the RDFs for the Ta-series 1T-like ternary TMDs, which demonstrate a transition from 1T to 1T′ character with increased proportion of high-Z chalcogenide. The peak at 3.4 Å, corresponding to M-M distances in the first coordination shell, develops a shoulder with the addition of Se to TaS_2–*δ*_Se_*δ*_. As the transition progresses from Se to Te in TaSe_2–*δ*_Te_*δ*_, the shoulder separates into a distinct peak 4.5 Å. The distortion pattern involves the separation of metal layers along the *b* axis, leading to distinct M-M distances, as shown in Fig. 1b. A higher ratio of the first-to-second peak indicates fewer regions of distortion, while a larger separation represents increased magnitude of distortion between the regions. A Jupyter notebook demonstrating M-M RDF analysis, called MM_distance_analysis.ipynb, is included in the scripts folder.

**Fig. 7:**
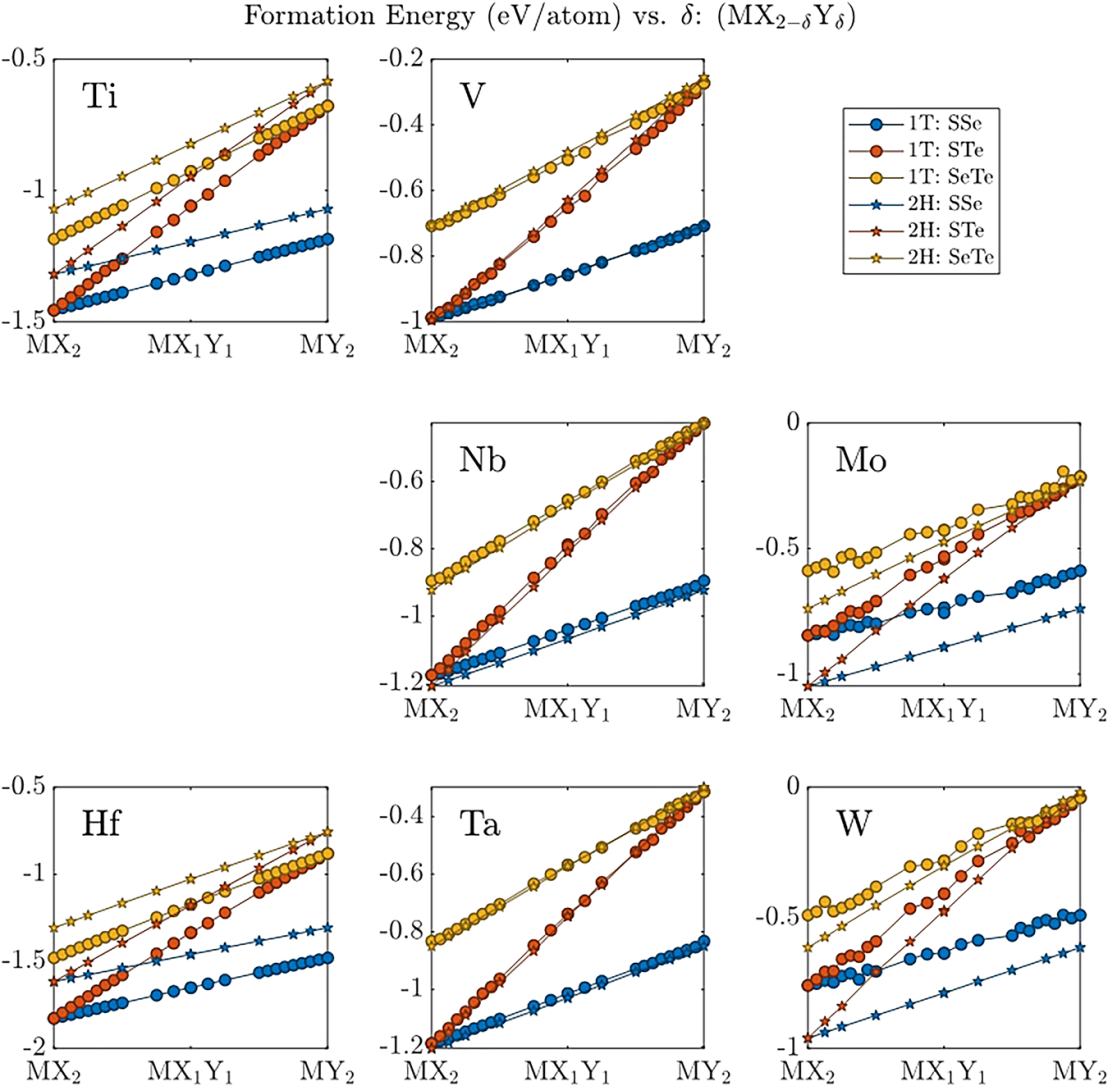
Formation energy vs. *δ* for ternary TMDs in MXY_DB. Formation energy is in units of eV/atom. The location of each plot corresponds to the location of its transition metal on the periodic table. The lighter chalcogenide is denoted as X, while the heaver chalcogenide is denoted as Y.

The formation energy of each structure was calculated via Eq. (1) using energetics of the relaxed structures in VASP_structural_relaxations along with the elemental energies in VASP_elemental. The raw values are included in info.csv under the Eform column and, for convenience, are plotted in Fig. 7. All systems in MXY_DB have negative formation energies, indicating that the reactions to form them from elemental precursors are exothermic. All ternary systems in MXY_DB show a near linear increase in formation energy with an increase in the proportion of heaver chalcogenide (*δ* → 2 in MX_2–*δ*_Y_*δ*_). Ternary TMDs with group 4 transition metals (Ti and Hf) show the 1T polytype to be more stable (~0.2 eV/atom) than the 2H polytype, while the opposite trend is observed for group 6 transition metals (Mo and W). Group 5 transition metals show near degeneracy of the 1T and 2H polytypes, with V having a slight preference for 1T and Nb and Ta having a slight preference for 2H. These differences between groups are caused by interactions between the increasingly occupied transition metal *d* shells and the valence chalcogen *p* electrons as the transition metal changes from group 4 to 6.

**Fig. 8:**
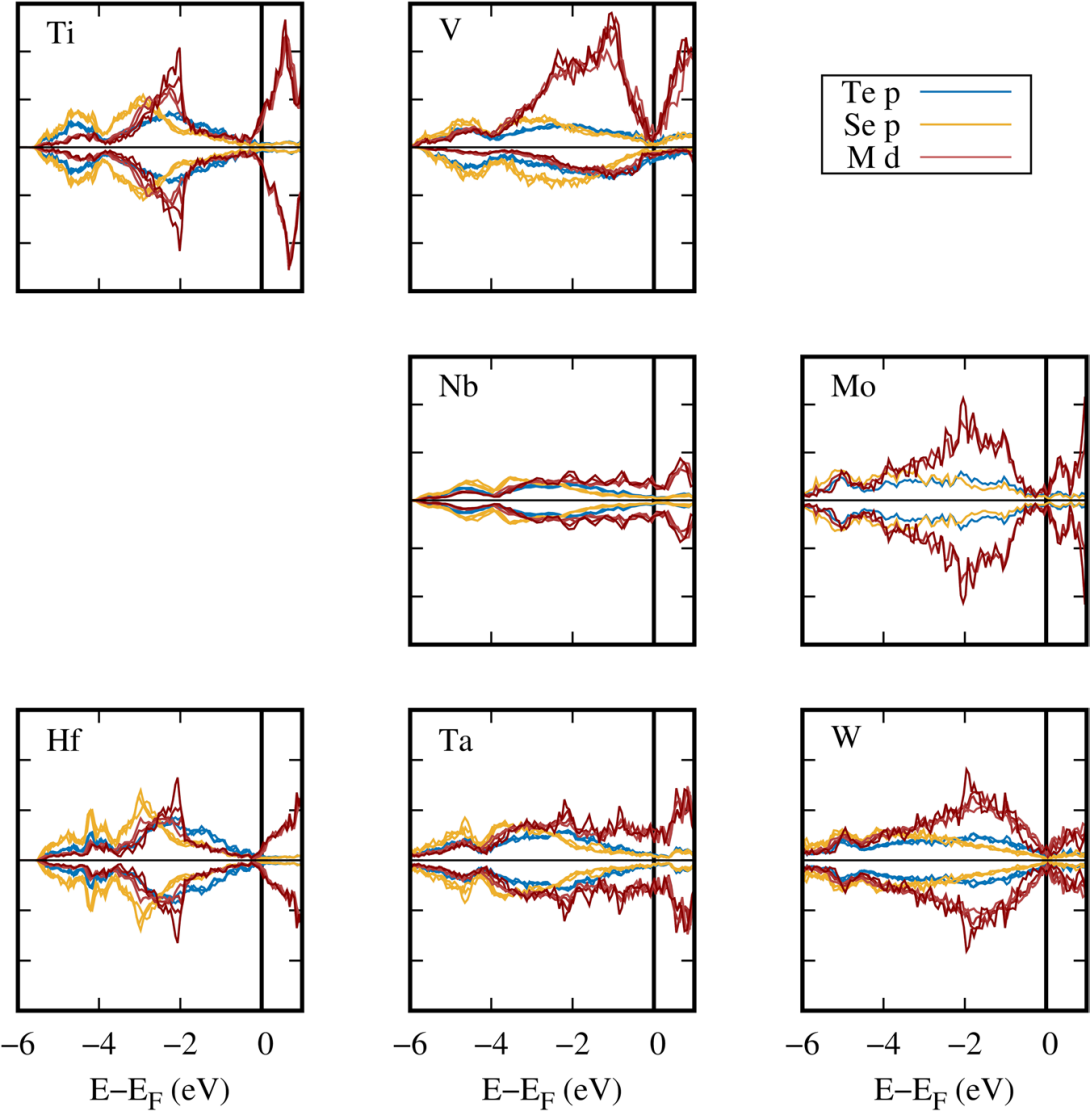
Electronic DOS of MSe_2–*δ*_Te_*δ*_ for all *δ*. The electronic DOS is projected onto chalcogenide *p* (Te in blue and Se in yellow) and transition metal *d* (red) orbitals.

As an illustration of the utility of MXY_DB for analyzing compositional trends in material properties, we plot the electronic density of states (DOS) of MSe_2–*δ*_Te_*δ*_ in Fig. 8. From this comparison, we see qualitatively different magnetic behaviors: there is spin polarization for M = V and not otherwise. Other interesting trends appear as the transition metal is varied: metal *d* hybridization with the chalcogen *p*-derived orbitals is much more pronounced for the 4- and 5*d* metals compared to the 3*d* metals, an effect we attribute to the larger *d* orbital radius, which results in increased spatial overlap with the valence *p* orbitals. The latter are split into two crystal field subbands the separation of which ranges from quite distinct (Hf, Ti, V) through modestly distinct (Nb, Ta) to barely discernible (W). The metal *d* DOS consists of components that share the same shape as the Te and Se upper crystal field valence subbands and excess states near the Fermi level. This behavior reflects covalent mixing and atomic-like occupation, respectively. The *d* orbitals of V adopt a local spin moment driven by onsite *d*-*d* exchange interactions. An interesting feature is that the total *d* occupation on the V sites varies with the doping level *δ*. Hence the existence of spin polarization can be adjusted by choice of transition metal, while the strength of the polarization can be controlled through the stoichiometry of the chalcogenide sublattice. The DOS at the Fermi level is also determined by the competition between onsite atomic effects and covalent interactions with the chalcogen layers resulting in a range of predicted metallicities from n-type semiconducting (Ti, Hf, Mo), through semimetallic (V, W), to metallic (Nb, Ta). We point out that these trends do not follow simple *d*-orbital filling arguments for M^4+^ transition metals that are commonly invoked^13^. The wide variety of electronic structures presented by the ternary TMDs in MXY_DB provides a foundation for the further analysis of electronic trends resulting from tuning chalcogenide stoichiometries. Considering large regions of composition space consistently clarifies systematic physical effects that can be missed by studies that focus on only one material.

MXY_DB is an open-source database of computed ternary TMD structures composed of 1T and 2H polytypes of seven transition metals (Ti, V, Nb, Mo, Hf, Ta, and W) with varying binary combinations of chalcogenides (S, Se, and Te). The database provides relaxed structures from which more detailed energetic analyses can be performed, a source for further data mining of the interesting structural properties present in ternary TMDs, and samples for training neural network potentials or other machine learning algorithms, among other functions invented in the creative researcher’s mind. Ternary TMDS with a single chalcogenide component and two transition metal components and quaternary TMDs containing mixtures of three different chalcogenides are not included in this database, but represent possible directions for future computational examination by the methods described in this report. The database is openly available at 10.6084/m9.figshare.21308157^46^.

## Data Availability

Scripts used to collate the ASE database and perform structural analyses are located alongside the dataset. All scripts are written in Python (v3.9) and are described in the accompanying README.md.
